# A molecular detection approach for a cotton aphid-parasitoid complex in northern China

**DOI:** 10.1038/s41598-019-52266-7

**Published:** 2019-11-01

**Authors:** Yu-Lin Zhu, Fan Yang, Zhi-Wen Yao, Yue-Kun Wu, Bing Liu, Hai-Bin Yuan, Yan-Hui Lu

**Affiliations:** 1 College of Plant Protection, Jilin Agricultural University, Changchun, 130118 China; 2 State Key Laboratory for Biology of Plant Diseases and Insect Pests, Institute of Plant Protection, Chinese Academy of Agricultural Sciences, Beijing, 100193 China

**Keywords:** Ecosystem services, Entomology

## Abstract

Aphid-parasitoid interactions have been widely used as a model system in research studies on the structure and functions of arthropod food web. Research on aphid-parasitoid food webs is hindered by their micromorphological characteristics and the high amount of labor associated with their development. Species-specific primers for cotton aphids and their parasitoids were designed and integrated into two multiplex PCRs and six singleplex PCRs, and all PCRs were optimized to achieve high specificity and sensitivity (100–10,000 DNA copies). One cotton aphid (*Aphis gossypii*) as well as three primary parasitoid and seven hyperparasitoid species or genera were detected using this molecular approach. This group comprises all the primary parasitoids and 97.2–99.6% of the hyperparasitoids reported in cotton fields in northern China. A tritrophic aphid-primary parasitoid-hyperparasitoid food web was then established. The described method constitutes an efficient tool for quantitatively describing the aphid-primary parasitoid-hyperparasitoid food webs and assessing the efficiency of the biological control of parasitoids in cotton fields in northern China.

## Introduction

The cotton aphid *Aphis gossypii* is highly abundant in cotton fields in northern China, and natural enemies play a vital role in the biological control of cotton aphids. For decades, much attention has been paid to the species composition, behavior and habits of the predatory enemies of aphids and the interactions between aphids and their predators^1–4^, but few studies have investigated aphid parasitoids in cotton fields due to the difficulty in identifying parasitoids and assessing aphid-parasitoid interactions. Presently, the cotton aphid and parasitoid composition in China can only be inferred from several previous references. Most of the recorded parasitoid species belong to Aphidiidae and can only be classified to the genus level^5–8^. In 2017, Yang *et al*. provided a relatively comprehensive description of the cotton aphid and parasitoid species composition in northern China, which includes three primary species and ten hyperspecies^9^. Due to the extremely small body and morphological characteristics of parasitoids, the aphid-parasitoid interactions and the effect of the biological control effect of parasitoids on aphids in fields cannot be clearly demonstrated. Only descriptions of field-collected parasitoid species, the parasitism rates^1,4^ and the mechanisms through which various factors, such as the CO_2_ concentration^10^ and aphid density^11,12^, influence the control efficiency of the control of specific parasitoid species under controlled conditions are available. For most researchers, accurate morphological identification remains difficult^13–16^, which restricts further progress in revealing aphid-parasitoid interactions in natural habitats.

Currently, DNA molecular detection techniques offer a new method for exploring and analyzing complex aphid-parasitoid food web relationships. Diagnostic PCR enables the recognition of species based on the bands produced by the electrophoresis of PCR products^17^. Species-specific primers targeting the 18S rRNA gene have been designed to distinguish two parasitoids, namely, *Lysiphlebus testaceipes* and *Lipolexis scutellaris*, from their host aphid species, namely, *Toxoptera citricida* and *Aphis gossypii*^18^. A 307-bp, species-specific COI sequence can be used to evaluate the parasitism rates of *Psyttalia concolor* and *Psyttalia lounsburyi* on the olive fly, *Bactrocera oleae*^19^. The parasitism level of the pomegranate aphid *Aphis punicae* can be estimated using 16S rDNA Aphidiinae-specific primers^20,21^. These results have demonstrated that the PCR approach can achieve higher accuracy in parasitism detection and species identification compared with rearing or dissection^18,19,21^. More recently, multiplex PCR was adopted for the detection of multiple species within one reaction. One multiplex PCR allows detection of the DNA of *Aphis fabae*, *Lysiphlebus testaceipes* and *Demetrias atricapillus* and thus provides a rapid and valid method for studying aphid-parasitoid-predator interactions^22^. The hyperparasitism and multiparasitism rates of *Pachyneuron siphonophorae* and *Syrphophagus aphidivorus* on the primary parasitoids *Aphelinus certus* and *Aphidius colemani* were previously detected through two multiplex PCRs^23^. Prior to the development of multiplex PCR, multiple trophic interactions, such as aphid-primary parasitoid, parasitoid-predator or parasitoid-parasitoid interactions, could not be obtained through direct observation, rearing or even singleplex PCR.

A molecular analysis through a combination of singleplex and multiplex PCRs has provided a complete picture of the parasitoid community in winter wheat fields in the UK, which includes nine primary species and two hyperspecies^24^. This combination combines the advantages of the high sensitivity and specificity of singleplex PCR with the ability to simultaneously detect multiple species or interactions through multiplex PCR. This method has been successfully used to compare the parasitism, hyperparasitism and multiparasitism levels between conventional and organic wheat fields, and the results revealed no significant differences^25^. Here, based on findings obtained in a previous study on the species composition of cotton aphid parasitoids^9^, we present a molecular detection approach that facilitates the identification of cotton aphids and parasitoids and the illustration of aphid-primary parasitoid-hyperparasitoid tritrophic interactions in cotton fields in northern China. A tritrophic food web was quantitatively described to provide a glimpse of the aphid-parasitoid interactions in the cotton fields. The results of this study will improve the current understanding of the efficiency of the biological control of each parasitoid species in cotton aphid populations and will allow the development of reasonable and valid management strategies.

## Results

### Multiplex and singleplex PCRs

**Figure 1:**
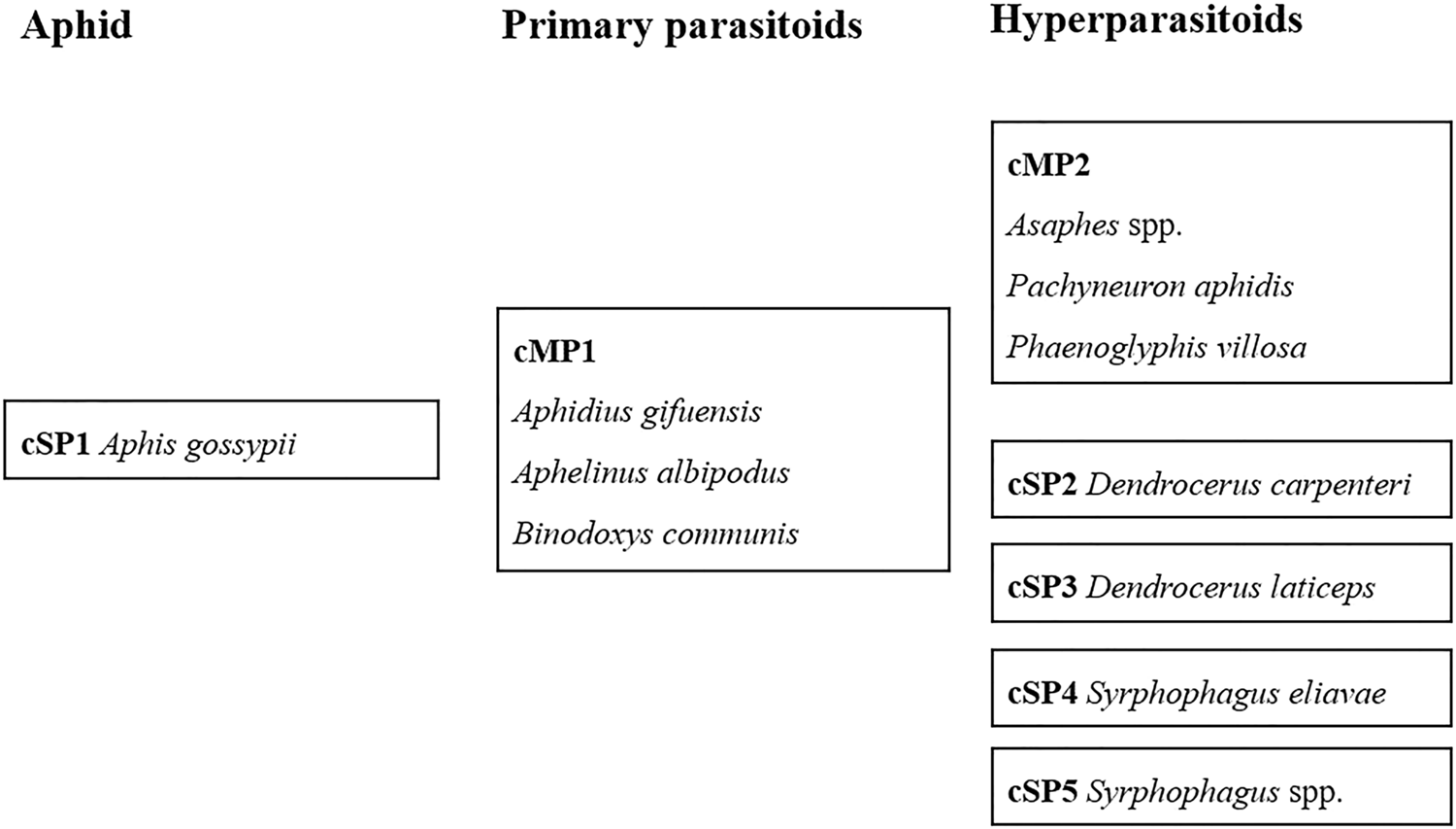
Composition of the cotton multiplex PCR detection system and the aphid and parasitoid species detected in each PCR.

To guarantee specificity and balance the sensitivity of the whole system, the multiplex PCR for hyperparasitoids was split into one multiplex PCR and several singleplex PCRs (Fig. 1). For the host, cSP1 detected the *Aphis gossypii* with an amplicon of 182 bp. The primary species was determined with cMP1 which contained three pairs of primers for *Aphidius gifuensis* (156 bp), *Aphelinus albipodus* (224 bp) and *Binodoxys communis* (374 bp) respectively. One multiplex PCR, cMP2 and four singleplex PCRs, cSP2-5 were used to detect the hyperparasitoids, involving *Asaphes* spp., *Pachyneuron aphidis*, *Phaenoglyphis villosa*, *Dendrocerus carpenteri*, *D. laticeps*, *Syrphophagus eliavae* and *Syrphophagus* spp. The size of all the amplicons in the detection system were below 425 bp and over 50 bp different in one multiplex PCR which ensured the effectiveness in the amplification and distinguishment (Table 1). Targeting on the 16 S gene, the species-specific primers were designed for the cotton aphid and two primary parasitoids, *Aphelinus albipodus* and *Binodoxys communis*. While, COI was the target gene for the rest pairs of species-specific primers (Table 2). The *Asaphes* spp. primers can detect *Asaphes suspensus* and *A. vulgaris*. The COI sequences of *Syrphophagus aphidivorus*, *Syrphophagus* sp. and *S. taeniatus* cannot be distinguished from each other in this system because only one pair of primers was generated for these three hyper-species. This group of detected insects contains all of the primary parasitoids and 97.2–99.6% of the hyperparasitoids reported in northern China^9^.

**Table 1:** GenBank accession, size and DNA detection limits for the amplification of cotton aphid and parasitoid species.

PCRs	Species	Accession no.	Size (bp)	Sensitivity (Copy number)	
				Single DNA template	Mixed DNA template
cSP1	*Aphis gossypii*	MG582182.1	182	500	
cMP1	*Aphidius gifuensis*	MF101669.1	156	100	500
	*Aphelinus albipodus*	MG581975.1	224		
	*Binodoxys communis*	FJ024082.1	374		
cMP2	*Asaphes* spp.	MF101678.1	173	10	100
	*Pachyneuron aphidis*	MF101673.1	328		
	*Phaenoglyphis villosa*	MF101672.1	413		
cSP2	*Dendrocerus carpenteri*	MF101674.1	100	500	
cSP3	*Dendrocerus laticeps*	MF101675.1	137	1000	
cSP4	*Syrphophagus eliavae*	MK370052.1	243	1000	
cSP5	*Syrphophagus* spp.	MK370053.1	425	10000

**Table 2:** Primers used in the cotton aphid-parasitoid multiplex PCR system.

PCR	Species	Forward (F) and reverse (R) primers (5′-3′)	Conc. (μM)	Target genes
cSP1	*Aphis gossypii*	F: TTGAATGAAAGATTTGATGAGAAATAG	1.0	16S
		R: TCACCCCAATAAAATAAATTTTAATT		
cMP1	*Aphidius gifuensis*	F: ATTAGGTTTATCTATAAGATTATTAATTCGG	0.4	COI
		R: TTACCAAATCCACCAATTATGATG		
	*Aphelinus albipodus*	F: ACATGGTTTTTTGATTATAATTTAAAATT	0.4	16S
		R: AAATTCTATAGGGTCTTCTCGTCTTTA		
	*Binodoxys communis*	F: AATAATATTAAGTCAAATCTGCCCAT	0.4	16S
		R: CCCTAAGGTAATTTATTTTAAAATTCC		
cMP2	*Asaphes* spp.	F: CAATGAATTTTAAATAGCTGCAGTATC	0.2	16S
		R: GGGTCTTCTCGTCTTTTAATTAAATA		
	*Pachyneuron aphidis*	F: GGATTTGGAAATTATTTAATTCCTATAT	0.2	COI
		R: TTGCTCATGCAAATAAAGGAATAA		
	*Phaenoglyphis villosa*	F: TAATATTATCAGCACCAGATATAGCG	0.2	COI
		R: TCCTATAGGRTCAAAAAAAGAAGTATTTAT		
cSP2	*Dendrocerus carpenteri*	F: CCCTCACTAATTTTACTTATCAATAGAATG	1.0	COI
		R: CAGCGTGTCTTAGATTAGACGTTAAG		
cSP3	*Dendrocerus laticeps*	F: GGGTCAATTAATTTTCTGTCAACTT	1.0	COI
		R: GCCCCAGCTAAAACGGGA		
cSP4	*Syrphophagus eliavae*	F: GAAGATGATCAAGTATATAATTGTATTGTAATC	1.0	COI
		R: TCAACATGTCCCAGTACCTTCC		
cSP5	*Syrphophagus* spp.	F: AATTGAATTATTTAAATTTTTTATAAATAATACAC	1.0	COI
		R: GCGGGTTAACTGGAATCATA		

#### Sensitivity assessment

The detection limit of the singleplex PCRs for *Aphis gossypii* was found to equal 500 DNA copies. The other singleplex PCRs could detect their target species (hyperparasitoids) with 500–10,000 DNA copies. The sensitivities of the same multiplex PCR for all tested species were set to the same level. The detection limit of cMP1with the single-species template was 100 copies, whereas that with the mixed species template was found to equal 500. In cMP2, ten DNA copies are sufficient for the detection of individual species (Table 1).

#### Specificity assessment

The specificity of each PCR was tested using related aphid and parasitoid species (see Fig. S1). Only the target species were amplified, which confirmed the high specificity of all the PCRs. The final concentration of each primer in cMP1 was 0.4 μM, cMP2 0.2 μM and all singleplex PCRs 1.0 μM (Table 2).

### Field sample screening and aphid-parasitoid food web construction

**Figure 2:**
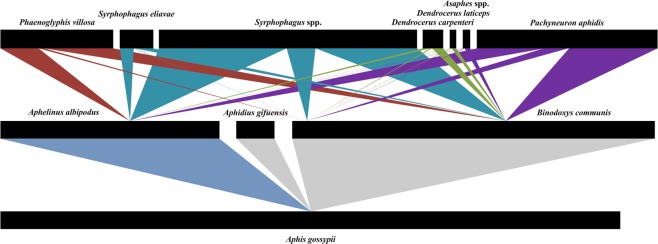
The tritrophic quantitative food web of aphids, parasitoids, and hyperparasitoids in cotton fields. The lower bars represent the aphid species; the middle bars represent the primary parasitoid species; and the upper bars represent the hyperparasitoid species. The links show the direct interactions between two species. The links for the hyperspecies from the same family have the same color.

Through cSP1, a total of 3158 mummified aphids were confirmed as valid samples, and 2192 of these samples were positive for primary parasitoid DNA. All three primary parasitoids, namely, *Aphidius gifuensis*, *Aphelinus albipodus* and *Binodoxys communis*, were detected in the mummified samples through cMP1 (Fig. 2). The most abundant primary parasitoid species was *B. communis*, and DNA for this species detected in 58.60% (1483) of the primary parasitized samples. The detection rates of *A. albipodus* and *A. gifuensis* were 35.30% (892) and 6.10% (155), respectively. Among all the primary parasitized aphids, 15.10% were found to contain the DNA of more than one primary parasitoid.

Further detection revealed that 843 mummified aphids contained DNA from seven hyperparasitoid species or genera. The three primary parasitoid species interacted with all the hyperparasitoids (Fig. 2). *Syrphophagus* spp. was detected in the largest proportion of samples (41.56%). *Pachyneuron aphidis* (29.17%) and *Phaenoglyphis villosa* (18.20%) were the second and third most abundant hyperspecies detected, respectively. The proportions of the remaining species or genera were less than 10%: *Asaphes* spp. (1.25%), *Dendrocerus carpenteri* (3.32%), *Dendrocerus laticeps* (1.10%) and *Syrphophagus eliavae* (5.40%). More than one species was detected in a single sample, and the proportion of multi-hyperparasitism was 26.93%.

## Discussion

In this study, a molecular detection approach consisting of two multiplex PCRs and six singleplex PCRs was established for the detection of one aphid, three primary parasitoid and seven hyperparasitoid species or genera. We previously determined the cotton aphid-parasitoid species composition in northern China through morphological identification^9^, but this manuscript describes the first study to determine the aphid and parasitoid species and reveal the aphid-parasitoid food web by using mummified aphids and a molecular detection method combining singleplex and multiplex PCRs. A similar molecular detection method was previously used to detect the predation of carabid beetles, *Demetrias atricapillus*, on parasitized aphid hosts^22^, and endoparasitism of the grain aphid *Sitobion avenae* by 11 parasitoid species was previously detected^24^. Two multiplex PCRs were also used to detect primary parasitoids and hyperparasitoids in the melon aphid, *A. gossypii*, and the detection results revealed trophic relationships that were not uncovered using the morphological method^23^. An effective molecular method was previously developed for the detection of 24 primary parasitoids and 16 hyperparasitoids appearing in wheat fields in central Europe^26^, but the proposed molecular approach could not be applied in our study due to the different research backgrounds and questions.

In this study, the COI mitochondrial and 16S rRNA genes were selected as the target genes for species-specific primer design. The COI mitochondrial gene has the most abundant sequence database and is thus widely used in invertebrate species identification^27^. 16S rRNA is a reliable gene for group-specific primer design^17^ and has been used in studies on species identification^28–31^. Several pairs of primers were initially designed, but only the best species-specific primers identified after sensitivity and specificity assessments were adopted for use with this detection system (Table 1). The efficiency of three genes as DNA markers was discussed by Ye *et al*. (2017b)^32^. COI is best suited for the design of primers for studies of Aphidiinae species, whereas 16S rRNA and 18S rRNA are most suitable for the design of primers for aphelinid primary parasitoids and hyperparasitoids and for the design of group-specific primers, respectively^32^. In our case, not all the species produced sequences with COI or 16S rRNA universal primers (Table S1). Genes, COI and nuclear long-wavelength rhodopsin (LWRh) were able to identify 50 species of Aphidiinae, but no single gene was able to distinguish all the species. Therefore, multiple target genes should be used to fully reveal the complex aphid-parasitoid composition^27,29^.

The cotton aphid-primary parasitoid-hyperparasitoid food web was obtained with the optimized detection approach (Fig. 2). All the hyperspecies or genera had links with the three primary species. The generalism of hyperparasitoids were also reported aphid-parasitoid complexes in wheat^22^, alfafa^33^ and potato^34^ which may be explained by the polyphagy of hyperparasitoids^34^. Compared with morphological identification, molecular detection yielded a similar species composition but different proportions of each parasitoid. The analysis of the primary species, *B. communis* was the most common in the parasitoid guild (58.60%), but more *A. gifuensis* (6.10%) and *A. albipodus* (35.30%) individuals were recovered using this method than by rearing (*A. gifuensis*: 0.3%, *A. albipodus*: 6.5%). In addition, 15.10% of mummified aphids contained two or three primary parasitoids. Intraguild competition might explain the low emergence of *A. gifuensis* and *A. albipodus* from the mummified aphids. Among the hyperparasitoids, *Syrphophagus taeniatus*, *S. aphidivorus* and *Syrphophagus sp*. accounted for 66.8–77.8% of all the hyperspecies obtained by rearing^9^, and *Syrphophagus* spp. was the most abundant (41.56%) species obtained through molecular detection. The proportion of *P. aphidis* increased from 10.0–19.5% to 29.17% through the use of the molecular approach^9^, and that of *P. villosa* increased from 0.2–6.3% to 18.20%. The remaining species showed slight differences in terms of composition^9^. We failed to extract the correct sequences for *Alloxysta* species, which accounted for a small portion of the hyperspecies (0.4–2.8%)^9^; and these species should thus be further examined in the future. However, no *Dendrocerus carpenteri* specimens were found by rearing^9^, and we surprisingly found that this species accounted for 3.32% of the hyperspecies in the molecular detection results. This result revealed the superiority of the molecular detection approach. This approach reveals not only hidden trophic interactions but also cryptic species.

Regarding aphid-parasitoid food web interactions, there are still many interesting topics worth exploring and discovering. A quantitative analysis of a 16-member wheat aphid-parasitoid food web revealed that lower agricultural intensification (AI) increased the parasitism rates and aphid abundance, whereas higher AI led to a more complicated food web and a more diverse aphid and parasitoid community^35^. The parasitism rates of *Lysiphlebus testaceipes* on wheat aphids exhibited the strongest correlation with landscape variables at a 3.2 km radius surrounding the wheat fields, and the parasitism rate increased with increases in the landscape diversity^36^. However, these researchers found that the parasitism rates and species richness of parasitoids were positively related to extensive and diversified non-crops in landscapes, but not to the scale of filed landscapes. The results obtained from a 2-4-year study also suggested that landscape management exerts a fluctuating effect on biological control of parasitoids^37^. Contradictory to results obtained in a previous study, a more recent study found a lower parasitoid population in complex landscapes with low agricultural intensification^38^. Hyperparasitoids reportedly prevent the biological control of primary parasitoids due to their substantial influence on parasitoid mortality^23^. Hyperparasitoids, as the higher trophic natural enemies, responded more sensitively than primary parasitoids to intensive farming pratices^39^. Our detection approach could be used to further depict the ecological services of primary parasitoids within different landscapes or the ecological disservices of hyperparasitoids.

DNA metabarcoding combined with next-generation sequencing (NGS) is the focus of much researches on feeding relationships^40–42^ and host-parasitoid interactions^43–45^. This approach revealed a higher parasitism rate in host eggs and larvae of the millet head miner, *Heliocheilus albipunctella*, in Senegal and two cryptic parasitoid species^45^. The large number of reads and the lack of prior knowledge regarding species composition make NGS an excellent method for exploring food web relationships. Information on the aphid and parasitoid species composition was acquired in this study, and the proposed molecular detection approach might be more convenient and easier to use than a method involving the sequences produced by NGS.

Here, we presented a molecular detection approach that combines singleplex and multiplex PCR techniques. The method accurately detects cotton aphid parasitoid species and establishes a quantitative food web of the aphids, parasitoids, and hyperparasitoids (Fig. 2) in northern China. Using this method, the question of how agricultural practices and landscape structure affect aphid-parasitoid interactions can be answered through the quantitatively analysis of food webs, and this information will facilitate the development of management strategies for the biological control of parasitoids.

## Materials and Methods

### DNA extraction

The DNA from the aphid and parasitoid specimens was extracted using a nondestructive method^9^ to ensure the integrity of the parasitoid body for morphological confirmation. The DNA of aphids was extracted using the DNeasy Blood and Tissue Kit (Qiagen, Germany) following the manufacturer’s recommended protocol. The Chelex DNA extraction method was adopted for the extraction of DNA from batches of mummified aphids. Field-collected mummified aphids were stored individually in 95% ethanol at −20 °C. The samples were taken out in advance to prevent the influence of ethanol on the DNA extraction efficiency. One individual mummified aphid was placed in one 1.5 mL centrifuge tube and crushed with a disposable sterile pestle. A mixed solution containing 150 μL of Chelex solution (10%), 20 μL of PBS solution (pH = 7.2) and 30 μL of proteinase K solution (10 mg/mL) was also added to each centrifuge tube. After gentle vibration and centrifugation, the tubes were transferred into a mental bath and maintained at 56 °C for 8–12 h and 96 °C for 20 min.

All DNA samples were stored at −20 °C for further detection. To avoid contamination, one or two negative controls were used in each DNA extraction. Negative controls with no insect specimens were analyzed using the standard extraction protocol.

### Primer design

Universal primers targeting the COI mitochondrial gene and 16S rDNA gene^9^ were used to obtain the standardized sequences of aphids and parasitoids (for species and sequence information, see Table S1). The trimmed sequences from the same gene were aligned using BioEdit Sequence Alignment Editor (version 7.2.5; Ibis Therapeutics, Carlsbad, CA, USA). The unique bases of the target species were selected to design the primers. For establishment of the multiplex PCR, multiple primer pairs were designed for each species. The amplicon sizes of the different species in one reaction differed by at least 50 bp. The primer sequences included in the system are listed in Table 2.

### Multiplex and singleplex PCRs

The cotton aphid-parasitoid multiplex PCR detection system developed in this study consists of five singleplex PCRs and two multiplex PCRs in (Fig. 1). The first singleplex PCR aims to detect *A. gossypii* and therefore to ensures the success of mummified aphid DNA extraction. Subsequently, a multiplex PCR, cMP1, can detect all three species of primary parasitoids, namely, *Aphidius gifuensis*, *Aphelinus albipodus* and *Binodoxys communis*. Another multiplex PCR, cMP2, involves three pairs of primers that can detect *Asaphes* spp., *Pachyneuron aphidis* and *Phaenoglyphis villosa*. The remaining four singleplex PCRs aim to detect *Dendrocerus carpenteri*, *D. laticeps*, *Syrphophagus eliavae* and *Syrphophagus* spp.

The singleplex PCR system (10 μL) included 1.5 μL of double-distilled H_2_O, 5.0 μL of 2 × Multiplex PCR Master Mix, 1.0 μL of the forward primer (10 μM), 1.0 μL of the reverse primer (10 μM) and 1.5 μL of the DNA template. For the multiplex PCRs, the 10-μL reaction system of cMP1 was composed of 2.5 μL of double-distilled H_2_O, 5.0 μL of 2 × Multiplex PCR Master Mix, 1.0 μL of the primer mix and 1.5 μL of the DNA template, whereas the 2.5 μL of double-distilled H_2_O in the cMP1 reaction system was replaced by 2.0 μL double-distilled H_2_O and 0.5 μL BSA (10 μg/μL) in the cMP2 reaction system. All the PCRs were performed using the following protocol: initial denaturation at 95 °C for 15 min; 35 cycles of 94 °C for 30 s, the annealing temperature for 90 s and 72 °C for 60 s; and a final extension for 10 min. The annealing temperatures for cSP1–5 and cMP1-2 were 61 °C, 62 °C, 63 °C, 58 °C, 60 °C, 63 °C and 61 °C, respectively.

#### Sensitivity assessment

To prepare the DNA templates, the target species were amplified with COI or 16 S universal primers. The PCR products were cleaned with a QIAquick PCR Purification Kit and then mixed with the solution provided with the Quant-iT™ PicoGreen® dsDNA Assay Kit for quantification following the recommended protocol. The DNA copy numbers of the templates were calculated with a DNA calculator (University of Innsbruck, Austria). The single-species or mixed-species templates were subjected to gradient dilution in TE buffer (pH of 8.0), and the diluted templates were used for sensitivity detection. In the same reaction, the detection limits of all the species were adjusted to the same level (Table 1).

#### Specificity assessment

The specificity of the multiplex and singleplex PCRs was assessed using the aphid and parasitoid templates (Fig. S1). Nonspecific amplification was blocked by the addition of bovine serum albumin (BSA, 10 μg/μL) and tetramethylammonium chloride (TMAC, 5 M) solution. Excpet the rare specimens, two replicates of each species were set as the templates in the specificity detection. The only amplification in the target species confirmed the specificity of primer pairs. The sensitivity of each PCR was then checked and optimized.

### Field sampling and mummified aphid screening

The aphid and parasitoid specimens used in the specificity and sensitivity assessments were collected in cotton fields in northern China or from storage at the University of Innsbruck, Austria. Three cotton fields (15 × 15 m^2^, var. SGK321) were planted in late April 2015–2017 at the Langfang Experimental Station, Chinese Academy of Agricultural Science (CAAS) (GPS coordinates: 116.6°E, 39.5°N). The mummified aphids were collected from the center of each cotton field (at least 5 m from the field boundary) from late May to the middle of September every five days and individually stored in centrifuge tubes filled with 95% ethanol at −20 °C. A total of 471, 2080 and 607 mummified aphids were collected in 2015, 2016 and 2017, respectively. During the sampling period, regular farming practices, such as fertilization (375 kg/ha urea, 225 kg/ha diammonium phosphate (DAP), and 150 kg/ha potassium sulfate, with DPC application 1 week after seedling emergence), tillage and weeding, were performed, and no insecticides were applied to the fields.

The collected samples were screened using the optimized multiplex PCR system. One negative and one positive PCR control were added to each 96-well PCR, and negative controls for DNA extraction were also included. The positive results from each sample were recorded for data analysis.

### Food web analysis

The bipartite package of R software (R Development Core Team 2016) was employed to establish the quantitative aphid-primary parasitoid-hyperparasitoid food web. The length of black bars refers to the amount of each species detected in the field-collected samples using this approach. The links represent the detected interactions between different trophic levels, and the links in same color indicate that the linked hyperparasitoids belong to the same family.

## Supplementary information

Supplementary information
