# Supplementary material for: A molecular detection approach for a cotton aphid-parasitoid complex in northern China
Source: Sci Rep. 2019 Nov 1;9:15836. doi: 10.1038/s41598-019-52266-7 (PMC6825200; doi:10.1038/s41598-019-52266-7)
Supplement: Supplementary file 1 — Supplementary information [file 41598_2019_52266_MOESM1_ESM.doc]

# For submission to *Scientific Reports*

**A molecular detection approach for a cotton aphid-parasitoid complex in northern China**

Yu-Lin Zhu^1,2*^, Fan Yang^2*^, Zhi-Wen Yao^2^, Yue-Kun Wu^2^, Bing Liu^2^, Hai-Bin Yuan^1^, Yan-Hui Lu^2^

^1^Agricultural College, Jilin Agricultural University, Jilin 130118, China. ^2^State Key Laboratory for Biology of Plant Diseases and Insect Pests, Institute of Plant Protection, Chinese Academy of Agricultural Sciences, Beijing 100193, China.

*These two authors contributed equally to this paper. Correspondence and requests for materials should be addressed to Y.H.L. (email: yhlu@ippcaas.cn).

**Table S1** The target genes and GenBank accessions of sequences from species used to design the cotton aphid-parasitoid multiplex PCR system

| **Species** | **Target Genes** | | **GenBank Accession** | |
| --- | --- | --- | --- | --- |
|  | **COI** | **16S** | **COI** | **16S** |
| *Aphis craccivora* Koch | × | × |  |  |
| *Aphis gossypii* Glover | × | × |  | MG582182.1 |
| *Macrosiphoniella yomogifoliae* (Shinji) | × | × |  |  |
| *Megoura viciae* Buckton | × | × |  |  |
| *Metopolophium dirhodum* (Walker) | × | × | MF040668.1 |  |
| *Rhopalosiphum padi* (Linnaeus) | × | × | MF101665.1 |  |
| *Schizaphis graminum* (Rodani) | × | × | MF101666.1 |  |
| *Sitobion avenae* (Fabricius) | × | × | MF101667.1 |  |
| *Uroleucon gobonis* (Matsumura) | × | × |  |  |
| *Aphelinus albipodus* Hayat and Fatima | | × |  | MG581975.1 |
| *Aphidius ervi* Haliday | × | × |  |  |
| *Aphidius gifuensis* (Ashmead) | × | × | MF101669.1 |  |
| *Aphidius uzbekistanicus* Luzhetzki | × | × |  |  |
| *Binodoxys communis* (Gahan) |  |  | FJ024082.1 |  |
| *Praon barbatum* Mackauer |  | × |  |  |
| *Praon volucre* (Haliday) |  | × |  |  |
| *Lysiphlebus fabarum* (Marshall) | | × |  |  |
| *Trioxys asiaticus* Telenga |  | × |  |  |
| *Phaenoglyphis villosa* (Hartig) | × | × |  |  |
| *Alloxysta consobrina* (Zetterstedt) |  | × |  |  |
| *Alloxysta fracticornis* (Thomson) | × | × |  |  |
| *Alloxysta japonicus* (Ashmead) | × | × |  |  |
| *Alloxysta pusilla* (Kieffer) | × |  |  | MF092874.1 |
| *Alloxysta victrix* (Westwood) |  | × |  |  |
| *Alloxysta xanthopsis* (Ashmead) |  |  |  | AY745772.1 |
| *Phaenoglyphis villosa* (Hartig) | × | × | MF101672.1 |  |
| *Dendrocerus carpenteri* (Curtis) | × | × | MF101674.1 |  |
| *Dendrocerus laticeps* (Hedicke) | × | × | MF101675.1 |  |
| *Asaphes suspensus* (Nees) | × | × |  | MF101678.1 |
| *Asaphes vulgaris* Walker | × | × |  | MF101679.1 |
| *Pachyneuron aphidis* (Bouché) | × | × | MF101673.1 |  |
| *Syrphophagus aphidivorus (Mayr)* | × |  | MK370053.1 |  |
| *Syrphophagus eliavae* sp.n. | × |  | MK370052.1 |  |
| *Syrphophagus* sp*.* | × |  | MK370053.1 |  |
| *Syrphophagus taeniatus* (Förster) | × |  | MK370053.1 |  |

Note: “×” means the obtained sequences of corresponding genes.

**Figure S1** Electrophoretograms for PCR specificity assessment


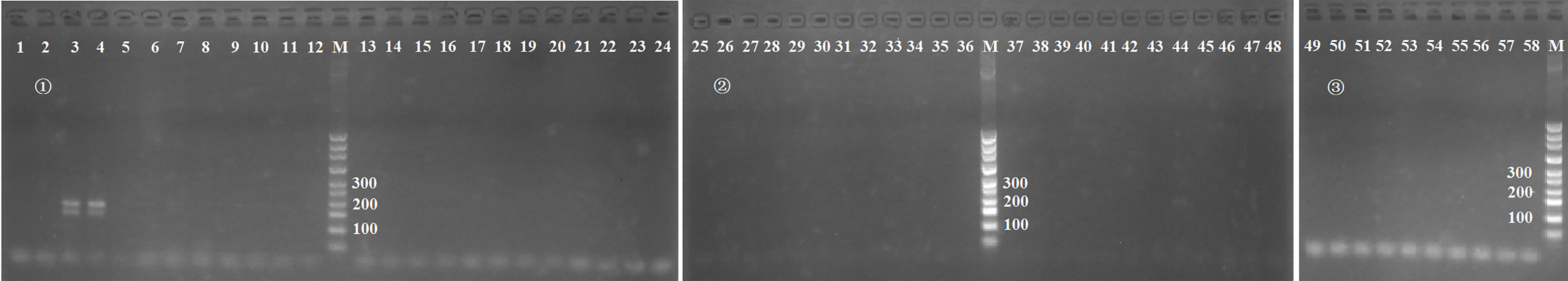


1. Electrophoretogram for cSP1 specificity assessment.

Notes: ① and ② are two parts from the same gel, and ③ is from another gel. 1,2 Negative control, 3,4 *Aphis gossypii* Glover, 5,6 *Aphis craccivora* Koch, 7,8 *Acyrthosiphon gossypii* Mordviko, 9,10 *Metopolophium dirhodum* (Walker), 11,12 *Sitobion avenae* Fabricius, 13,14 *Schizaphis graminum* (Rodani), 15,16 *Rhopalosiphum padi* L., 17,18 *Aphelinus albipodus* Hayat and Fatima, 19,20 *Binodoxys communis* (Gahan), 21,22 *Aphidius gifuensis* Ashmead, 23,24 *Praon barbatum* Machauer, 25,26 *Lysiphlebus fabarum* (Marshall), 27,28 *Trioxys asiaticus* Telenga, 29,30 *Praon volucre* (Haliday), 31,32 *Aphidius uzbekistanicus* Luzhetski, 33,34 *Aphidius ervi* Haliday, 35,36 *Asaphes suspensus* (Nees), 37,38 *Asaphes vulgaris* Walker, 39,40 *Pachyneuron aphidis* (Bouché), 41,42 *Phaenoglyphis villosa* (Hartig), 43,44 *Alloxysta brevis* (Thomson), 45,46 *Alloxysta pusilla* (Kieffer), 47,48 *Syrphophagus aphidivorus* (Mayr), 49,50 *Syrphophagus eliavae* Japoshvili, 51,52 *Syrphophagus* sp., 53,54 *Syrphophagus taeniatus* (Förster), 55,56 *Dendrocerus carpenteri* (Curtis), 57,58 *Dendrocerus laticeps* (Hedicke), M: DNA marker (50 bp DNA ladder, Biomed, Beijing, China).


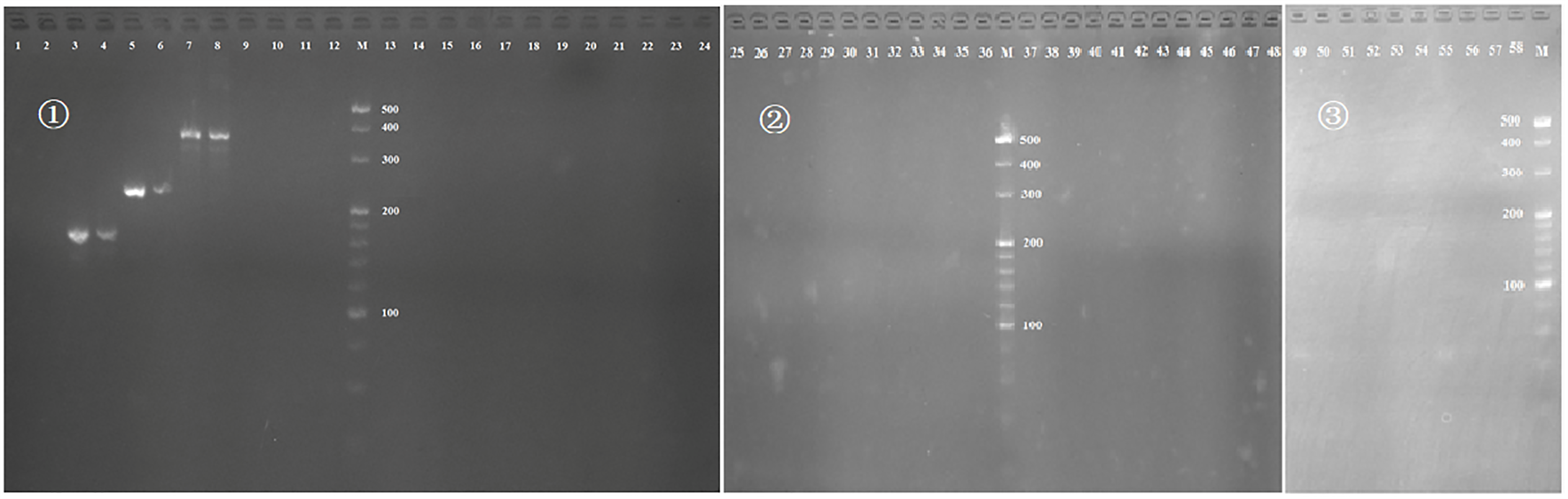


1. Electrophoretogram for cMP1 specificity assessment.

Notes: ①, ② and ③ are from different gels. 1,2 Negative control, 3,4 *Aphidius gifuensis* Ashmead, 5,6 *Aphelinus albipodus* Hayat and Fatima, 7,8 *Binodoxys communis* (Gahan), 9,10 *Praon barbatum* Machauer, 11,12 *Lysiphlebus fabarum* (Marshall), 13,14 *Trioxys asiaticus* Telenga, 15,16 *Praon volucre* (Haliday), 17,18 *Aphidius uzbekistanicus* Luzhetski, 19,20 *Aphidius ervi* Haliday, 21,22 *Asaphes suspensus* (Nees), 23,24 *Asaphes vulgaris* Walker, 25,26 *Pachyneuron aphidis* (Bouché), 27,28 *Phaenoglyphis villosa* (Hartig), 29,30 *Alloxysta brevis* (Thomson), 31,32 *Alloxysta pusilla* (Kieffer), 33,34 *Syrphophagus aphidivorus* (Mayr), 35,36 *Syrphophagus eliavae* Japoshvili, 37,38 *Syrphophagus* sp., 39,40 *Syrphophagus taeniatus* (Förster), 41,42 *Dendrocerus carpenteri* (Curtis), 43,44 *Dendrocerus laticeps* (Hedicke), 45,46 *Metopolophium dirhodum* (Walker), 47,48 *Sitobion avenae* Fabricius, 49,50 *Schizaphis graminum* (Rodani), 51,52 *Rhopalosiphum padi* L., 53,54 *Aphis gossypii* Glover, 55,56 *Aphis craccivora* Koch, 57,58 *Acyrthosiphon gossypii* Mordviko, M: DNA marker (20 bp DNA ladder, Takara, Japan).


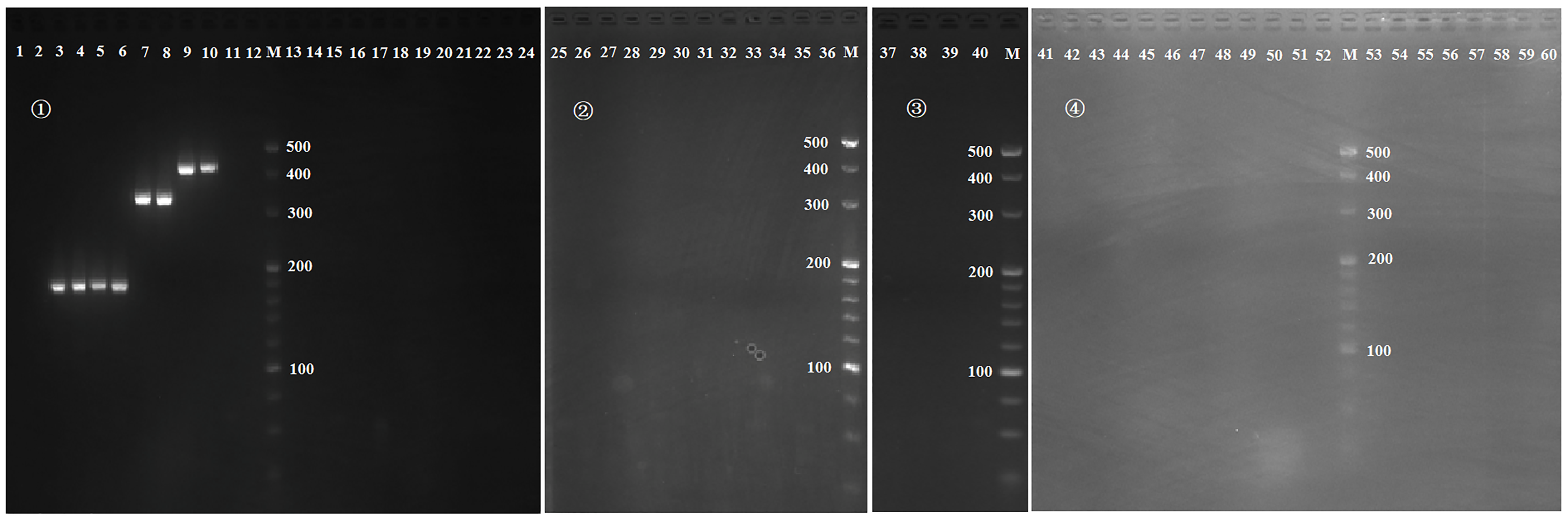


1. Electrophoretogram for cMP2 specificity assessment.

Notes: ①, ②, ③ and ④ are from different gels. 1,2 negative controls, 3,4 *Asaphes suspensus*, 5,6 *A. vulgaris*, 7,8 *Pachyneuron aphidis*, 9,10 *Phaenoglyphis villosa*, 11,12 *Praon volucre*, 13,14 *Aphidius uzbekistanicus*, 15,16 *A. ervi*, 17,18 *A. gifuensis*, 19,20 *Alloxysta* sp., 21,22 *Syrphophagus aphidivorus*, 23,24 *S. eliavae*, 25,26 *Syrphophagus* sp., 27,28 *S. taeniatus*, 29,30 *Dendrocerus carpenteri*, 31,32 *D. laticeps*, 33,34 *Metopolophium dirhodum*, 35,36 *Sitobion avenae*, 37,38 *Schizaphis graminum*, 39,40 *Rhopalosiphum padi*, 41,42 *Aphis gossypii*, 43,44 *Aphelinus albipodus*, 45,46 *Binodoxys communis*, 47,48 *Praon barbatum*, 49,50 *Lysiphlebus fabarum*, 51,52 *Trioxys asiaticus*, 53,54 *Alloxysta brevis*, 55,56 *Alloxysta pusilla*, 57,58 *Aphis craccivora*, 59,60 *Acyrthosiphon gossypii*, M, DNA marker (20 bp DNA ladder, Takara).


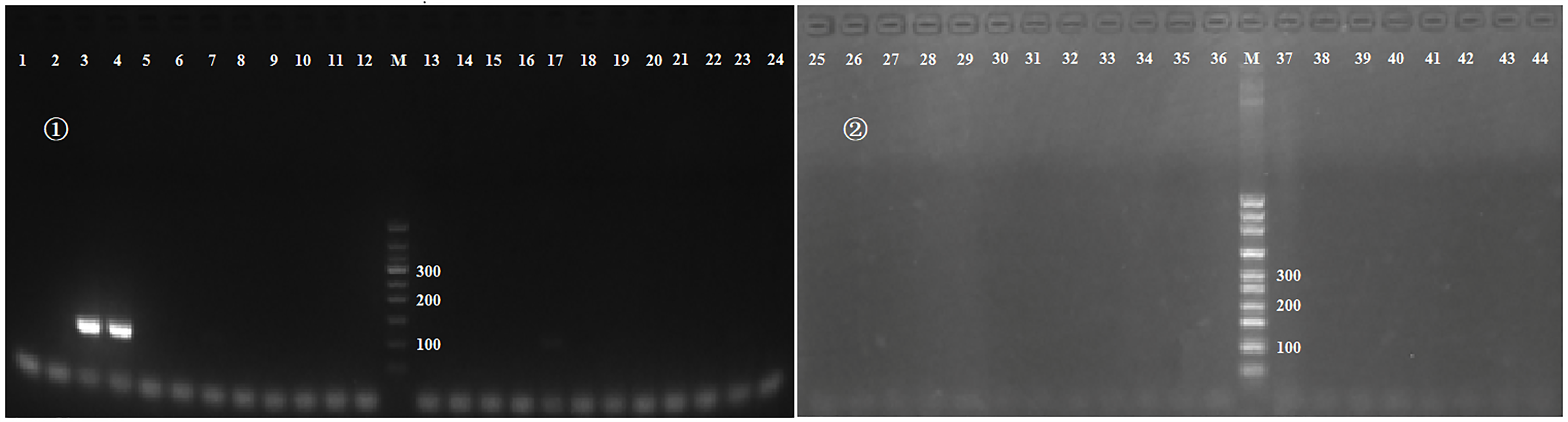


1. Electrophoretogram for cSP2 specificity assessment.

Notes: ① and ② are from two different gels. 1,2 negative controls, 3,4 *Dendrocerus carpenteri*, 5 *Aphidius ervi*, 6 *A. gifuensis*, 7 *Aphidius uzbekistanicus*, 8 *Praon volucre*, 9 *Schizaphis graminum*, 10 *Metopolophium dirhodum*, 11 *Sitobion avenae*, 12 *Rhopalosiphum padi*, 13 *Alloxysta* sp. 1, 14 *Alloxysta* sp. 3, 15 *Alloxysta* sp. 2, 16 *Phaenoglyphis villosa*, 17 *Syrphophagus aphidivorus*, 18 *S. eliavae*, 19 *Syrphophagus* sp., 20 *Syrphophagus taeniatus*, 21 *Pachyneuron aphidis*, 22 *D. laticeps*, 23 *Asaphes suspensus*, 24 *A. vulgaris*, 25,26 *Aphis gossypii*, 27,28 *Aphelinus albipodus*, 29,30 *Binodoxys communis*, 31,32 *Praon barbatum*, 33,34 *Lysiphlebus fabarum*, 35,36 *Trioxys asiaticus*, 37,38 *Alloxysta brevis*, 39,40 *Alloxysta pusilla*, 41,42 *Aphis craccivora*, 43,44 *Acyrthosiphon gossypii*, M, DNA marker (50 bp DNA ladder, Tiangen).


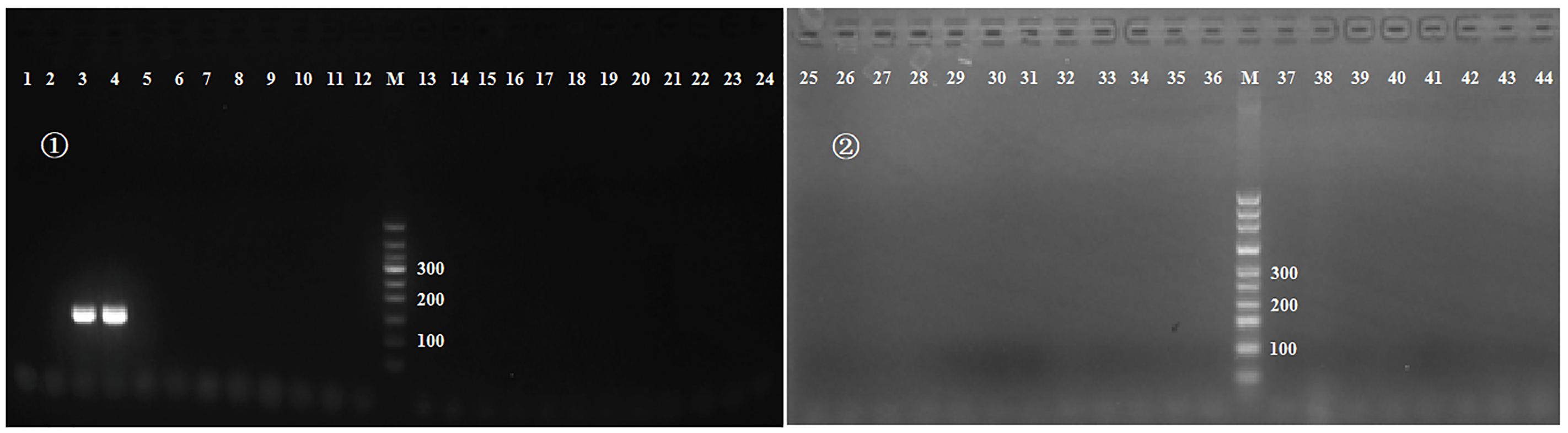


1. Electrophoretogram for cSP3 specificity assessment.

Notes: ① and ② are from two different gels. 1,2 negative controls, 3,4 *Dendrocerus laticeps*, 5 *Aphidius ervi*, 6 *A. gifuensis*, 7 *Aphidius uzbekistanicus*, 8 *Praon volucre*, 9 *Schizaphis graminum*, 10 *Metopolophium dirhodum*, 11 *Sitobion avenae*, 12 *Rhopalosiphum padi*, 13 *Alloxysta* sp. 1, 14 *Alloxysta* sp. 3, 15 *Alloxysta* sp. 2, 16 *Phaenoglyphis villosa*, 17 *Syrphophagus aphidivorus*, 18 *S. eliavae*, 19 *Syrphophagus* sp., 20 *Syrphophagus taeniatus*, 21 *Pachyneuron aphidis*, 22 *D. carpenteri*, 23 *Asaphes suspensus*, 24 *A. vulgaris*, 25,26 *Aphis gossypii*, 27,28 *Aphelinus albipodus*, 29,30 *Binodoxys communis*, 31,32 *Praon barbatum*, 33,34 *Lysiphlebus fabarum*, 35,36 *Trioxys asiaticus*, 37,38 *Alloxysta brevis*, 39,40 *Alloxysta pusilla*, 41,42 *Aphis craccivora*, 43,44 *Acyrthosiphon gossypii*, M, DNA marker (50 bp DNA ladder, Tiangen).


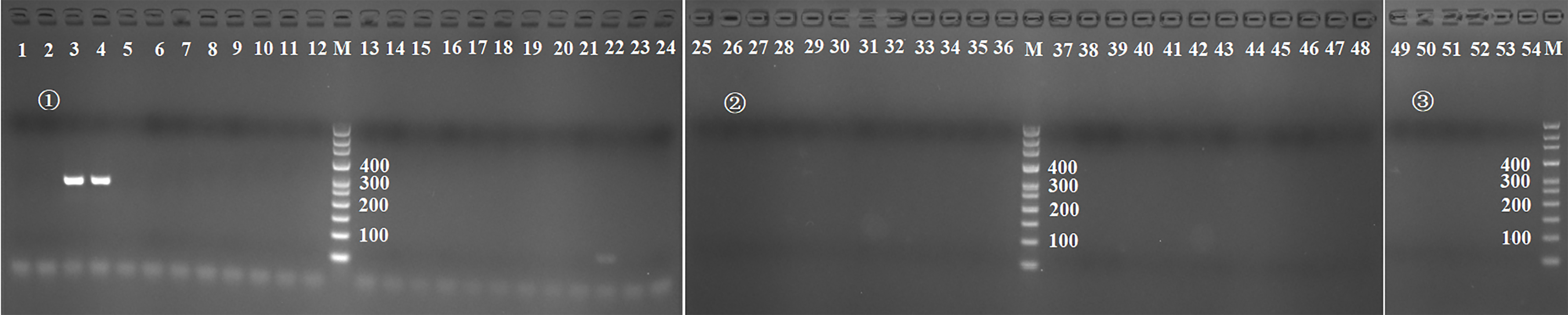


1. Electrophoretogram for cSP4 specificity assessment.

Notes: ① and ② are from the same gels, but ③ is from a different gel. 1,2 negative controls, 3,4 *Syrphophagus eliavae*, 5,6 Syrphophagus spp., 7,8 *Aphidius gifuensis*, 9,10 *Aphelinus albipodus*, 11,12 *Binodoxys communis*, 13,14 *Praon barbatum*, 15,16 *Lysiphlebus fabarum*, 17,18 *Trioxys asiaticus*, 19,20 *Praon volucre*, 21,22 *Aphidius uzbekistanicus*, 23,24 *Aphidius ervi*, 25,26 *Asaphes suspensus*, 27,28 *A. vulgaris*, 29,30 *Pachyneuron aphidis*, 31,32 *Phaenoglyphis villosa*, 33,34 *Alloxysta brevis*, 35,36 *Alloxysta pusilla*, 37,38 *Dendrocerus carpenteri*, 39,40 *Dendrocerus laticeps*, 41,42 *Metopolophium dirhodum*, 43,44 *Sitobion avenae*, 45,46 *Schizaphis graminum*, 47,48 *Rhopalosiphum padi*, 49,50 *Aphis gossypii*, 51,52 *Aphis craccivora*, 53,54 *Acyrthosiphon gossypii*, M, DNA marker (50 bp DNA ladder, Tiangen).


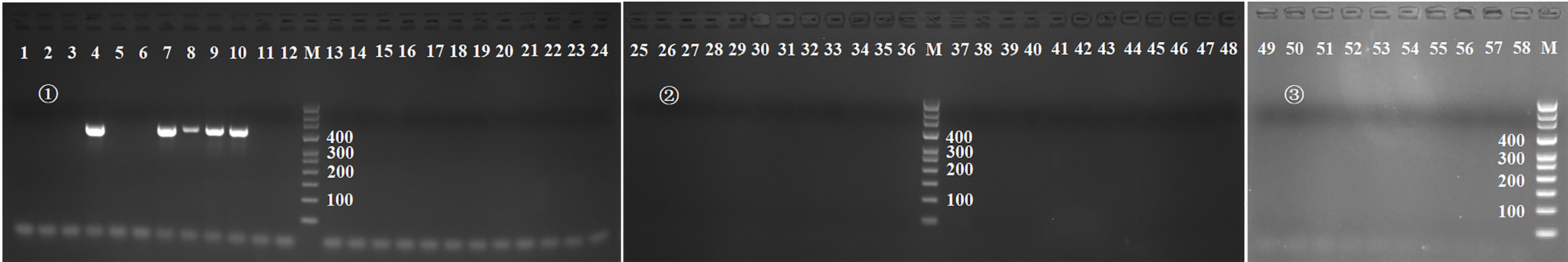


1. Electrophoretogram for cSP5 specificity assessment.

Notes: ① and ② are from the same gels, but ③ is from a different one. 1,2 negative controls, 3,5,6 *Syrphophagus eliavae*, 4 *Syrphophagus aphidivorus*, 7,8 *Syrphophagus* sp*.*, 9,10 *Syrphophagus taeniatus*, 11,12 *Aphidius gifuensis*, 13,14 *Aphelinus albipodus*, 15,16 *Binodoxys communis*, 17,18 *Binodoxys communis*, 19,20 *Lysiphlebus fabarum*, 21,22 *Trioxys asiaticus*, 23,24 *Praon volucre*, 25,26 *Aphidius uzbekistanicus*, 27,28 *Aphidius ervi*, 29,30 *Asaphes suspensus*, 31,32 *A. vulgaris*, 33,34 *Pachyneuron aphidis*, 35,36 *Phaenoglyphis villosa*, 37,38 *Alloxysta brevis*, 39,40 *Alloxysta pusilla*, 41,42 *Dendrocerus carpenteri*, 43,44 *Dendrocerus laticeps*, 45,46 *Metopolophium dirhodum*, 47,48 *Sitobion avenae*, 49,50 *Schizaphis graminum*, 51,52 *Rhopalosiphum padi*, 53,54 *Aphis gossypii*, 55,56 *Aphis craccivora*, 57,58 *Acyrthosiphon gossypii*, M, DNA marker (50 bp DNA ladder, Tiangen).
